# Clustered photoplethysmogram pulse wave shapes and their associations with clinical data

**DOI:** 10.3389/fphys.2023.1176753

**Published:** 2023-10-26

**Authors:** Serena Zanelli, Kornelia Eveilleau, Peter H. Charlton, Mehdi Ammi, Magid Hallab, Mounim A. El Yacoubi

**Affiliations:** ^1^ Laboratoire Analyse, Géométrie et Applications, University Sorbonne Nord, Villetaneuse, France; ^2^ Axelife, Saint-Nicolas-de-Redon, France; ^3^ Department of Public Health and Primary Care, University of Cambridge, Cambridge, United Kingdom; ^4^ Laboratoire Analyse, Géométrie et Applications, University of Sorbonne Nord, Saint-Denis, France; ^5^ Clinique Bizet, Paris, France; ^6^ SAMOVAR Telecom SudParis, CNRS, Institut Polytechnique de Paris, Palaiseau, France

**Keywords:** PPG, waveform, classification, machine learning, deep learning, unsupervised learning

## Abstract

Photopletysmography (PPG) is a non-invasive and well known technology that enables the recording of the digital volume pulse (DVP). Although PPG is largely employed in research, several aspects remain unknown. One of these is represented by the lack of information about how many waveform classes best express the variability in shape. In the literature, it is common to classify DVPs into four classes based on the dicrotic notch position. However, when working with real data, labelling waveforms with one of these four classes is no longer straightforward and may be challenging. The correct identification of the DVP shape could enhance the precision and the reliability of the extracted bio markers. In this work we proposed unsupervised machine learning and deep learning approaches to overcome the data labelling limitations. Concretely we performed a K-medoids based clustering that takes as input 1) DVP handcrafted features, 2) similarity matrix computed with the Derivative Dynamic Time Warping and 3) DVP features extracted from a CNN AutoEncoder. All the cited methods have been tested first by imposing four medoids representative of the Dawber classes, and after by automatically searching four clusters. We then searched the optimal number of clusters for each method using silhouette score, the prediction strength and inertia. To validate the proposed approaches we analyse the dissimilarities in the clinical data related to obtained clusters.

## 1 Introduction

The photoplethysmogram (PPG) signal contains precious information about the blood vessels and heart activity. The digital volume pulse (DVP) is defined as the portion of PPG signal corresponding to one cardiac cycle. In young individuals, the DVP exhibits clearly defined systolic and diastolic peaks. The diastolic peak is attenuated with increasing age (Dawber et al., 1973). The systolic peak is related to the forward pressure wave from the heart to the finger. The diastolic wave, also called the reflected wave, depends on the amount of reflection (due to muscular tone) in small arteries (Millasseau et al., 2006). DVP shape changes with age (Allen and Murray, 2003), blood pressure (Millasseau et al., 2006), atherosclerosis (Rozi et al., 2012), and other cardiovascular diseases such as arrhythmia (Sardana et al., 2021) and coronary artery disease (Saritas et al., 2019). DVP wave shapes vary between subjects and with the presence of pathologies. It can be used to assess a variety of cardiovascular properties, such as estimating blood pressure (Kurylyak et al., 2013), detecting diabetes (Zanelli et al., 2022), or assessing vascular ageing (Charlton et al., 2022). An understanding of typical DVP wave shapes could contribute to the physiological interpretation of wave shapes, and could help in the development of robust DVP wave analysis algorithms. Most of the DVP biomarkers extraction algorithms assume that DVPs have a standardized shape. However real DVPs can show more than one peak. In this case, the biomarker cannot be computed directly but a suitable pre-processing has to be applied to the wave in order to obtain an estimation. For our knowledge very few studies address the DVP shape morphology classification topic.

**FIGURE 1:**
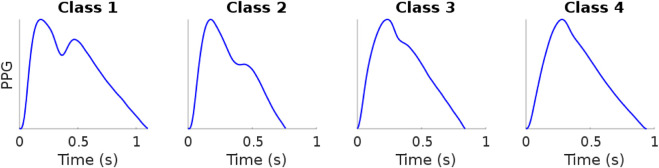
Example of digital volume pulse Dawber classes. Data sourced from Charlton et al. (2016). Figure: Classes of photoplethysmogram (PPG) pulse wave shape: Examples of the four classes of pulse wave shape proposed by Dawber et al. Reproduced form https://commons.wikimedia.org/wiki/File:Classes_of_photoplethysmogram_(PPG)_pulse_wave_shape.svg, licensed under CC-BY 4.0.

In 1973, Dawber et al., 1973 defined four classes of DVP shape based on the characteristics of the dicrotic notch (Figure 1). The four classes range from a visible and clearly marked dicrotic notch (Class 1) to a non visible dicrotic notch (Class 4). However, DVPs exhibit far more shape variations than are captured in the characteristics of the dicrotic notch. Other attempts have been made to identify typical DVP wave shapes: frequency analysis to classify the DVPs into three classes based on the age (Sherebrin and Sherebrin, 1990); machine learning and deep learning methods trained over handcrafted features to classify the DVPs shape into the four classes proposed by Dawber et al. (Tigges et al., 2016); and second derivative analysis used to obtain four DVP templates (Takada et al., 1996). Wang et al., 2013 proposed a multi-Gaussian fitting to classify DVPs into five classes. With respect to Dawber et al., they introduced an intermediate class where no notch develops but there is a notable reflected wave in the systolic component of the pulse wave. The main limitation of these studies is the pre-emptive choice of the number of DVP classes.

We used non-supervised approaches to identify clusters of DVP wave shapes as follows. First, we investigated different approaches for clustering DVP waves with the aim of identifying the best approach. K-medoids clustering was used to cluster DVP wave shapes based on: 1) handcrafted DVP features; 2) Derivative Dynamic Time Warping (DDTW) distances; or (iii) features extracted from a convolutional neural network autoencoder (CNN AE). K-medoids was used instead of K-means as it is less affected by outliers, and it guarantees that each medoid (the DVP shape representing the entire cluster) is an actual DVP (Park and June 2009). Second, we investigated whether the optimal number of clusters is four, as suggested by Dawber et al., or a different number. To do so, all the clustering methods were tested when the number of clusters was fixed to four (with and without fixing the medoids to DVP waves typical of Dawber’s four classes), and when the optimal number of clusters was determined through one of: the prediction strength method (Tibshirani and Walther, 2005); the silhouette score (Shahapure and Nicholas, 2020); or clusters inertia (Syakur et al., 2018). Third, we investigated whether any of the obtained clusters were clinically relevant. To do so, we analysed the related clinical data for each cluster to assess whether there were significant differences between clusters. The dataset used in this study contained approximately 11,000 DVPs from 300 subjects aged 20–80 years old. Our contributions can be summarized as follows.

• We clustered DVP waves using a K-medoids approach with three different feature sets. We compared the results obtained with 1) a dataset composed by fourteen PPG handcrafted features, 2) a dataset composed by DDTW pairwise distances and 3) a dataset composed by features automatically extracted from a CNN autoencoder.
• We tested the proposed approaches with four clusters to compare the obtained results with the Dawber et al. classes. Then, we investigated the optimal number of clusters using the silhouette score, inertia and the prediction strength methods. The approaches have been also tested by imposing four representative medoids, selected by a human expert.
• We investigate whether or not the obtained clusters are clinically relevant by analysing the distribution of the clinical data associated with each cluster.

## 2 Material and methods

### 2.1 Dataset

**FIGURE 2:**
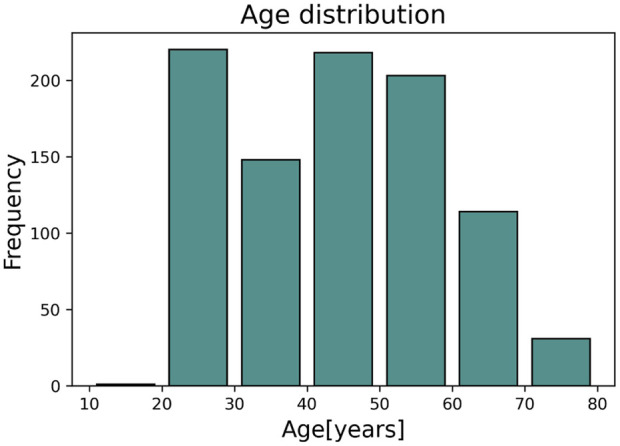
Age distribution of the used dataset.

The dataset used in this study contains PPG signals recorded from 300 different subjects, providing a total of about 11,057 DVPs. Table 1 presents the subject characteristics: the subjects ranged from 19 to 83 years old; and included normotensives, hypertensives and hypotensives. Figure 2 represents the age distribution of the dataset. The PPG signals were acquired at 1 kHz with the pOpmètre device (Axelife, France) (Obeid et al., 2017). pOpmètre is a medical device that measures the pulse wave velocity (PWV) between the finger and the toe in order to assess arterial stiffness. Prior to measurement, subjects were asked to lie down and rest for about 5 min. The device utilizes transmittance PPG with red and infrared light. Each measurement takes up to 14 s to be computed. Some subjects in the dataset had more than one measurement taken. Only the finger signals were used in this study. The quality assessment process described in (Zanelli et al., 2021) was used to select only the high quality parts of the signals. Signals were then segmented into DVPs. DVP waves were normalised in time (100 samples) and amplitude (between zero and one). The employed dataset is composed only of DVP waves without any other information related to the shape. Since no labels are available, we propose an unsupervised approach to cluster the waves, using three different DVP extracted features. This process is further explained in the next section.

**TABLE 1:** Clinical data. Mean, standard deviation, minimum and maximum values of the clinical data related to the used DVPs dataset.

Feature	Mean (*±* std)	Min	Max
**Age[years]**	44.31 ± 14.34	19.48	83.00
**Weight[kg]**	74.60 ± 16.99	45.00	180.00
**Height[cm]**	170.04 ± 8.45	140.00	196.00
**PWV[m/s]**	7.99 ± 2.91	3.70	26.10
**PAS[mmHg]**	124.77 ± 16.01	90.00	190.00
**PAD[mmHg]**	76.10 ± 9.73	48.00	120.00
**BMI[kg/m** ^**2**^ **]**	25.78 ± 5.12	6.00	56.00
**BPM[bpm]**	71.82 ± 14.89	40.00	185.00

The dataset was split into train and test sets using 70% and 30% of the available data respectively. Since one subject can have different DVP shapes along the same measurement, one subject can contribute to the train, validation or test set at the same time. The validation set is composed of 30% of the training test. The same train, validation and test sets were used with all the proposed methods in order to be able to compare the results. The train and validation sets were used to train the CNN AE while the test set was used for clustering.

### 2.2 Clustering pulse waves

The K-medoids technique was used to cluster DVP waves. K-medoids is a partitional algorithm firstly proposed in 1980 (Rdusseeun and Kaufman, 1987). Its objective is to split a dataset into *k* clusters by minimising the distance between the center of each cluster and the samples assigned to that cluster. The center of the cluster (also known as a medoid) is defined as the sample in the cluster whose average dissimilarity to all the remaining objects in the cluster is minimal. The chosen medoid is an actual sample of the dataset, in contrast to the k-means algorithm. Furthermore, because k-medoids minimizes the sum of dissimilarities between two samples of the dataset instead of the sum of squared euclidean distances, it is more robust to noise and outliers than k-means (Kaur et al., 2014). In this study, we applied the K-medoids in three ways, as now described.

### 2.2.1 Using handcrafted features

Clustering was performed using twenty one handcrafted features were extracted from the DVPs contained in the dataset. The features include those proposed in the literature to assess DVP morphology (Tigges et al., 2016), second derivative features (Mouney et al., 2021), and statistical shape features such as kurtosis and skewness. We performed the correlation analysis to identify and remove highly correlated features. This resulted in fourteen handcrafted features being selected, as reported in Table 2. After checking the feature distributions, we applied a logarithmic transformation to three of the remaining features. We then standardized the features by subtracting the mean and scaling to unit variance. The fourteen features were clustered using the K-medoids approach.

**TABLE 2:** DVPs features used for clustering with the handcrafted DVP feature approach. Abb: feature name abbreviation.

Feature	Abb	Description
Dicrotic-diastolic notch	DDR	Ratio between dicrotic notch and diastolic peak amplitude. Set to 1.1 if no diastolic point is detected
Relative downslope sum	RDS	Area under the curve from the maximum descendet slope to the end of the pulse
Downslope derivative mean	DDM	Mean value of the pulse derivative after the systolic peak
Skewness	Skew	Measure of the pulse asymmetry
Max second derivative	P_b_2Dev_A	First minimum amplitude of the second derivative
Index min second derivative	P_b_2Dev_i	Index of the first minimum amplitude of the second derivative
Min second derivative	P_a_2Dev_A	Maximum amplitude of the second derivative
Index max second derivative	P_a_2Dev_i	Index of the maximum amplitude of the second derivative
Integral of the curve	S_P_Onde	Total area under the curve
Number of peaks	nbr_peaks	Number of peaks inside the pulse
Logarithm of Rise time	log (RT)	Logarithm of the rise time
Logarithm of Kurtosis	log (Kurt)	Logartimic measure of the “tailedness”
Augmentation index	AI_bin	Augmentation index, binary variable. If augmentation index could be computed the variable has high value
Down slope derivative variance	log (DDV)	Logarithm of the variance of the pulse derivative variance during the down slope

### 2.2.2 Using dynamic time warping

Dynamic Time Warping (DTW) is an algorithm employed to estimate the similarity between two time series (Müller, 2007). DTW was first introduced around 1960 and applied in speech recognition around 1975 (Senin, 2008). Over the years, this algorithm has been demonstrated to be very effective in matching time series of all kinds (Bagnall et al., 2016), such as for handwriting classification (El-Yacoubi et al., 2019). It has already been applied to PPG and ECG signals in various fields to assess signal quality (Li and Clifford, 2012), identify fine finger gestures (Zhao et al., 2018), or for human verification systems (Hwang et al., 2021). We define two time series as *X* = (*x*_1_, *x*_2_, … , *x*_*n*_) and *Y* = (*y*_1_, *y*_2_, … , *y*_*m*_).

**FIGURE 3:**
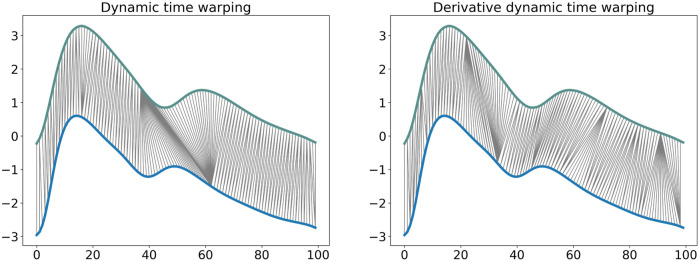
Dynamic time warping and derivative dynamic time warping comparison. It is observable how DDTW is able to match the same shape modifications with respect to the DTW that matches points that have low relative distance.

The DTW similarity measure is computed as the minimal cost of aligning the two time series as described in Algorithm 1 (Sakoe and Chiba, 1978). Several adaptations have been proposed to improve the efficiency and the effectiveness of this algorithm. Local constraints such as the Itakura parallelogram (Itakura, 1975) or the Sakoe-Chiba band (Sakoe and Chiba, 1978) have been found to reduce the computational complexity of the unconstrained DTW and also improve accuracy when used with a 1 Nearest-Neighbor (1-NN) classifier (Geler et al., 2019). We implemented DDTW using a SakoeChiba window of length *w* = 20 samples. DTW is likely to be successful when applied to two sequences that are similar except for local accelerations and decelerations in the time axis. However, in our case the DVPs differed mostly on the *Y*-axis. We found that DTW did not provide successful results, as the algorithm matched points with lower mutual distance rather than points with similar shapes. Therefore, we implemented Derivative Dynamic Time Warping (DDTW), in which the time series *X* and *Y* are substituted with their derivatives *X*′ and *Y*′. This takes into consideration the slopes of the DVPs in order to compute the minimal cost (Keogh and Pazzani, 2001). DVPs were further Z-normalised before performing DDTW measures. Figure 3 shows the application of classical DTW and DDTW. The DDTW similarities were clustered using the K-medoids approach.

Algorithm 1 Calculating the dynamic time warping (DTW) distance between time series.

 Require: n, m ≥ 0
  int DTW[0..n, 0..m]
  int i, j, cost
  s: array [1..n], t: array [1..m]
  **for** i ≔ 1 to n **do**
    **for** j ≔ 1 to m **do**
      DTW[i, j] ≔ infinity
    **end** **for**
  **end** **for**
  DTW[0, 0] ≔ 0
  **for** i ≔ 1 to n **do**
    **for** j ≔ 1 to m **do**
      cost≔ abs(s[i] - t[j])
      DTW[i, j] ≔ cost + minimum(DTW[i-1, j ], DTW[i , j-1], DTW[i-1, j-1])
    **end** **for**
  **end** **for**
  return DTW[n, m]

### 2.2.3 Convolutional neural network autoencoder

A Convolutional Neural Network (CNN) AutoEncoder model was used to automatically extract features from DVP waves (Chafik et al., 2019). CNNs are widely employed in biomedical signal processing (Alaskar, 2018) since they are capable of extracting features by exploiting the convolution operation between the input and learnt filters (Li et al., 2017). The autoencoder is trained to extract features from the input (performing a dimension compression step) and reconstruct it using the learnt features by minimising the Mean Squared Error (MSE) between the reconstructed input and the actual input. The model is composed of 6 convolutional layers and three dense layers. A flattening layer is added after the last convolution in order to reduce the dimensions and pass the feature maps to the fully connected layer. Relu activation functions inside CNN layers were used, while a sigmoid activation function was used for the reconstruction layer. The model architecture is represented in Table 3. We optimize the bottleneck layer size, the learning rate and *λ* L2 regularisation factor using Autonomio Talos python tool (Talos, 2019). The optimal model was chosen as a trade-off between validation loss and bottleneck layer size. A latent size of eight was chosen, indicating that eight features were extracted from DVP waves. This provided the smallest bottleneck layer size that performed well enough compared to the best validation loss obtained. Once the autoencoder is trained, only the encoder part is used to extract features from the DVPs. The eight features were clustered using the K-medoids approach.

**TABLE 3:** CNN AutoEncoder architecture.

Layers	Output shape
Encoder	
Input layer	(None, 100)
Convolution layer	(None,100,32)
MaxPooling	(None,50,32)
Convolution layer	(None,50,32)
MaxPooling	(None,25,32)
Convolution layer	(None,25,32)
MaxPooling	(None,13,32)
Flatten	(None,416)
Dense	(None,8)
Decoder	
Dense	(None,8)
Dense	(None,416)
Reshape	(None,13,32)
Convolution layer	(None,26,32)
Cropping	(None,25,32)
UpSampling	(None,50,32)
Convolution layer	(None,50,32)
UpSampling	(None,100,32)
Convolution layer	(None,100,32)
Flatten	(None,3,200)
Dense	(None,100)

### 2.3 Investigating the optimal number of clusters

We employed three different methods to investigate the optimal number of clusters: the silhouette score (Rousseeuw, 1987), the prediction strength (Tibshirani and Walther, 2005), and the cluster inertia (Syakur et al., 2018). These are now described.

The silhouette score is calculated by taking into account the mean intra-cluster distance *a*, and the distance between a sample and the nearest cluster that the sample is not a part of *b*. The silhouette score *s* for a sample is:

$$s=\left(b-a\right)/\max\left(a,b\right).$$ (1)

The score is then averaged over all samples. This score measures how well a dataset sample *i* matches the chosen clustering scheme. A score of 1 means the samples are correctly clustered, a score of 0 means the samples could belong to other clusters, and a score of −1 means that the cluster contains the wrong samples.

The prediction strength of the clustering *C*(*X*_*tr*_, *k*) is a defined as:

$$ps\left(k\right)=\underset{1\leq j\leq k}{\min}\frac{1}{n_{k,j}\left(n_{k,j}-1\right)}\;\underset{i\neq i^{\prime }\in A_{kj}}{\sum }D{\left[C\left(X_{tr},k\right),X_{te}\right]}_{ii^{\prime }},$$ (2)

where *n*_*k*,*j*_ is the number of observations in the *j*th cluster, *D*[*C*(*X*_*tr*_, *k*), *X*_*te*_] is the co-membership matrix of size (*X*_*tr*_ (train set), *X*_*te*_ (test set)) and *C*(*X*_*tr*_, *k*) is the clustering algorithm fitted to the training set. In other words, for each test cluster, the proportion of observation pairs in that cluster that are also assigned to the same cluster by the training set centroids is computed. The prediction strength is the minimum of this quantity over the *k* test clusters. The maximum number of clusters for which the prediction strength is above a certain threshold is then chosen. Although the experiments ran by the authors suggest 0.8–0.9 as a good value for the threshold, the latter may be interpreted on a case-by-case basis.

The cluster inertia was computed as follows:

$$\overset{N}{\underset{i=1}{\sum }}{\left(x_{i}-C_{k}\right)}^{2},$$ (3)

where *N* is the number of samples within the data set and *C* is the center of a cluster. It computes the sum of squared distance of each sample in a cluster to its cluster center.

We have to highlight that, although we tested three different methods to investigate the optimal number of clusters, the final choice of the optimal number of clusters is still affected by personal interpretation.

### 2.4 Investigating the clinical relevance of obtained clusters

The clinical relevance of the clustering was investigated as follows.

The Kruskal Wallis test (McKight and Najab, 2010) was used to assess whether there was a significant difference between the values of each clinical parameter between the clusters found using each clustering approach (template, baseline, and optimal). In the case of significant differences, the null hypothesis that the mean ranks of the groups are the same was rejected, and the Welch test (Alekseyenko, 2016) was used to identify the clusters for which the differences were significant.

Since significant differences were found between the clinical parameters of different clusters when using all the clustering approaches, we also conducted a more in-depth analysis of differences. Here, we propose a technique to identify which methods better discriminate the clinical data distribution among the clusters. Inspired by core shape modelling (Boudaoud et al., 2010), we assessed the intrinsic shape variations of the clinical data probability density functions (PDFs) across different clusters. This method assesses the dissimilarity between cluster PDFs by computing the distance between the relative reversed cumulative distribution function. The CSM objective is to remove the shape modification related to the *x*-axis shift and focus only on the shape. Differently from the original approach, we did not want to remove the *x*-axis shift. Thus, we computed the distances between the reversed cumulative distribution function taking into consideration the possible *x*-axis shift. Probability density functions (PDFs) of the clinical data related to the clusters were computed for each of the proposed methods. From the reversed cumulative distribution function it is now possible to compute the euclidean distance. This measure gives an index to quantify the similarity between two PDFs. The larger it is, the better the method is able to cluster waves in a clinical relevant manner. The averaged distance *d* that we propose is computed as follows:

$$d=\frac{1}{k\left(k-1\right)}\;\overset{k}{\underset{i=1}{\sum }}w_{i}\overset{k}{\underset{i\neq j}{\sum }}w_{j}dist\left(i,j\right)$$ (4)

where $w_{n}=\frac{N_{DVP_{n}}}{N_{\mathrm{DVP}}}$ is a weight that penalises clusters composed of fewer DVPs computed as the ration between the number of DVP contained in the cluster *n* and the total amount of DVP in the dataset and $dist\left(i,j\right)=|CDF_{i}^{-1}-CDF_{j}^{-1}|$ is the distance between two reserved cumulative distribution functions.

## 3 Results and discussion

In this section we present and discuss the obtained DVP clusters. First, we compare results obtained using the baseline approach and the template approach to understand whether the clusters of DVP waves are similar to Dawber’s classes. Second, we present the results obtained using each of the three methods for identifying the optimal number of clusters. Finally, we assess the clinical relevance of the obtained clusters.

Clusters were visualised using a nonlinear dimensionality reduction method: the t-Distributed Stochastic Neighbor Embedding (t-SNE) (Van der Maaten and Hinton, 2008). This method provides a faithful lower-dimensional representation where the distribution of the original data is conserved also in the lower dimension representation. It achieves this by modeling the dataset with a dimension-agnostic probability distribution, finding a lower-dimensional approximation with a closely matching distribution (Li et al., 2017). In order to visualise the clinical data among the different clusters we present, for each proposed method, a radar plot representing the clinical data normalized averages among the clusters. Figure 5, Figure 6 and Figure 7 respectively represent the t-SNE cluster representation, the medoids and the PPG waves attributed to each cluster; and the radar plot of the clinical parameters.

### 3.1 Comparing with Dawber’s classes

This section presents the results of clustering the dataset into four clusters using each of the three clustering methods: handcrafted features, CNN AE automated features, and the DDTW similarity matrix. We chose four clusters to allow comparison with the DVP shapes proposed by Dawber et al. In a first (baseline) approach each method was used to automatically identify the four clusters. In a second (template) approach, the medoids of the four clusters were imposed as DVP waves selected by an expert to correspond to the four classes identified by Dawber et al. We iteratively computed the assignments of the pulses to the clusters.

The results are shown in Figure 6, where the top row shows the template approach, and the middle row shows the baseline approach. Dawber’s classes (top row) were designed based on differences in dicrotic notch characteristics. The medoid DVP waves automatically identified in the DDTW baseline approach are most similar to Dawber’s classes: cluster 1 (corresonding to the youngest subjects as observed in Figure 7) exhibits a marked dicrotic notch (similar to Dawber’s class 1), which gradually disappears in older subjects (clusters 0, 2, and 3 respectively, corresponding to Dawber’s classes 2, 3, and 4) (see Figure 6). The clusters automatically identified when using the CNN autoencoder method are less similar to Dawber’s classes, and those identified when using handcrafted features are even less similar still.

The number of DVP waves in each cluster was less balanced when imposing Dawber’s classes as medoids than when automatically identifying medoids (see Table 4). For instance, when using handcrafted features, the proportion of DVP waves allocated to each cluster ranged from 3% to 43% when prescribing Dawber’s template classes, compared to 20%–33% when allowing cluster medoids to be identified automatically. There were similar imbalances when using CNN autoencoder features, and DDTW.

**TABLE 4:** Number of DVPs in each cluster and for each proposed method.

Features	Handcrafted	CNN	DDTW	Handcrafted	CNN	DDTW	Handcrafted	CNN	DDTW
Approach	Template			Baseline			Optimal		
Cluster 0	746	656	1,506	803	701	1,161	495	714	172
%	20	18	41	22	19	32	14	20	5
Cluster 1	1,230	1,602	247	920	854	1,087	584	351	601
%	34	44	7	25	23	30	16	10	16
Cluster 2	1,580	1,151	1,354	734	998	878	743	642	780
%	43	32	37	20	27	24	20	18	21
Cluster 3	87	234	536	1,186	1,090	520	606	342	345
%	2	6	15	33	30	14	17	9	9
Cluster 4	-	-	-	-	-	-	539	452	725
%							15	12	20
Cluster 5	-	-	-	-	-	-	676	579	635
%							19	16	17
Cluster 6	-	-	-	-	-	-	-	563	388
%								15	11
Total waves	3,643	3,643	3,643	3,643	3,643	3,646	3,643	3,643	3,646

The clusters identified using DDTW not only exhibited differences in dicrotic notch characteristics (similarly to Dawber’s classes) but also exhibit changes in the characteristics of the systolic portion of the DVP wave (see Figure 6): the systolic peak becomes wider with age (i.e., from cluster 1 to 0, 2, and 3), and the secondary systolic wave disappears with age. This secondary systolic wave could be a reflected wave caused by the elasticity of the artery (Luo et al., 2014) or the reflection of the forward wave at the renal artery branch (Nagasawa et al., 2022).

### 3.2 Determining the optimal number of clusters

In order to determine the optimal number of clusters, we computed the silhouette score, the inertia and the prediction strength of the proposed approaches (see Section 2.3). Whilst these methods do require some subjective interpretation, using multiple methods allowed us to reduce the level of subjectivity. The computational cost of applying the prediction strength to the DDTW matrix was found to be high resulting in an extremely long runtime (>10 days) requiring, in our opinion, a non-justifiable amount of resources (Patterson et al., 2021). Therefore, the silhouette score and inertia were used to select optimal numbers of clusters. Where available, the result was then compared with the prediction strength.

**FIGURE 4:**
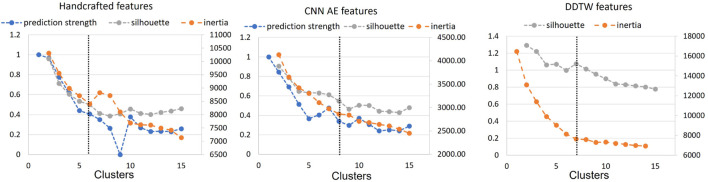
Prediction strength, inertia and silhouette score for clusters *k* = [1, 15] for handcrafted and CNN AE approach. Inertia and silhouette score for clusters *k* = [1, 15] for DDTW approach. Black dotted line: chosen number of clusters.

Figure 4 shows the results for the three approaches when varying the number of clusters from 1 to 15. Silhouette score and inertia have been used jointly to select the optimal number of clusters. We searched in the results a number of cluster k, for which the obtained silhouette score was close to 1 and the inertia presented an elbow. The selected number of clusters was then compared with the obtained prediction strength analysis. Based on these results, we chose not to use a threshold of 0.8 for the prediction strength because it would have been too restrictive. The optimal number of clusters was determined as 6, 7, and 7 clusters for the handcrafted feature, the CNN AE, and the DDTW approaches respectively.

### 3.3 Determining the optimal clustering method

**FIGURE 5:**
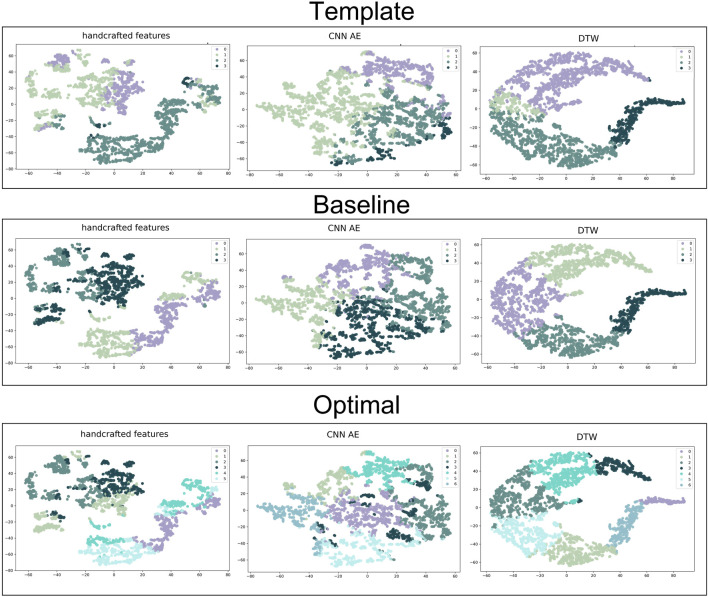
t-SNE clustering representation of all the proposed approaches. From the top to the bottom: template, baseline and optimal approach. From the left to the right: handcrafted, CNN AE and automated features.

**FIGURE 6:**
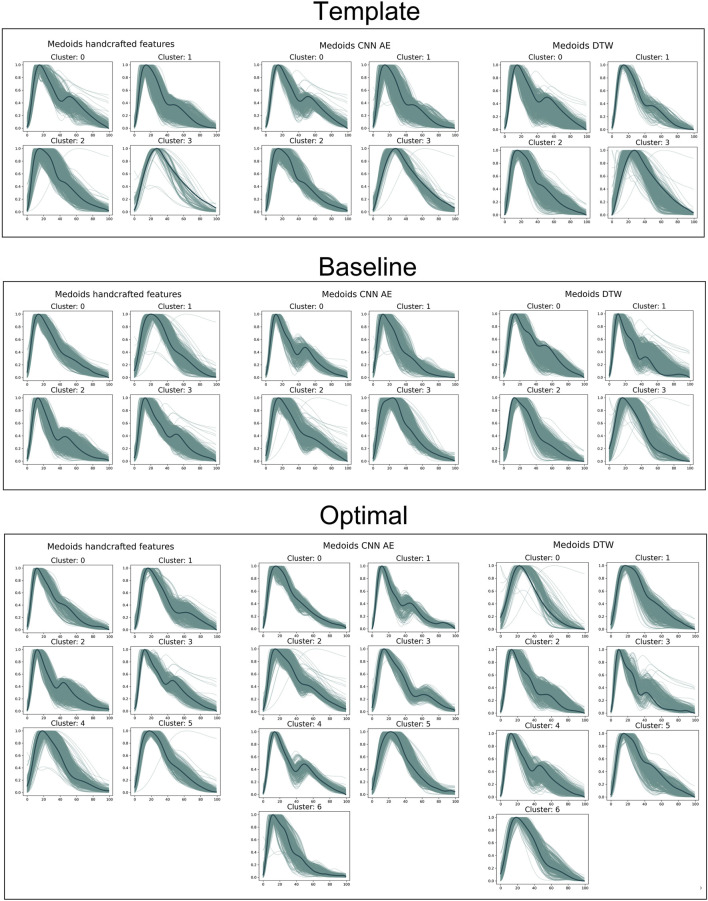
Medoids (dark green) and DVPs (light green) for all the proposed approaches. From the top to the bottom: template, baseline and optimal approach. From the left to the right: handcrafted, CNN AE and automated features.

The t-SNE visualisations (Figure 5) help determine which clustering approach best separates DVP waves into clusters. The DDTW approach appears to better separate the clusters when using the template, baseline, or optimal approaches. However, we can observe from the medoids plot (Figure 6) some clusters appear to be very similar in all of the three proposed approaches. We used the intra-cluster inertia to further investigate the performance of different clustering approaches (see Figure 4). The intra-cluster inertia was lowest for the DDTW approach (approximately 0.2), and substantially higher for the other approaches (approximately 0.4 for the CNN autoencoder approach, and 0.5 for the handcrafted features approach). Based on this, we suggest that the DDTW clustering approach performed best in this study.

### 3.4 Investigating the clinical relevance of clusters

**FIGURE 7:**
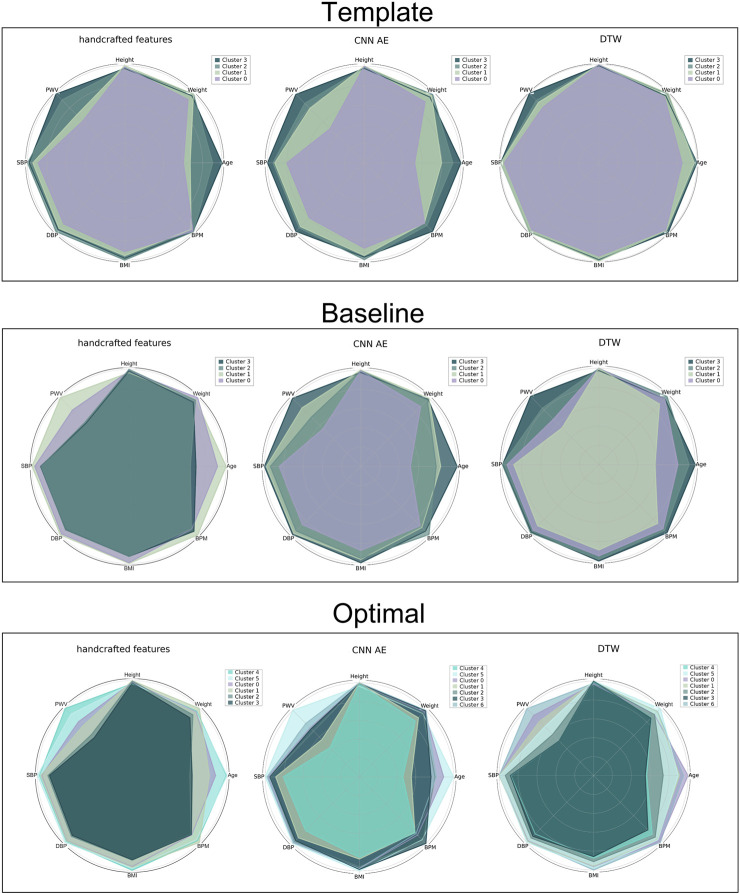
Radar plot for all the proposed approaches representing the average value of the clinical data related to the clustered DVPs. From the top to the bottom: template, baseline and optimal approach. From the left to the right: handcrafted, CNN AE and automated features.

Clustering a dataset composed of real DVPs with no prior information about the possible optimal number of clusters, can be challenging. The main difficulty is represented by the impossibility of validating a certain approach. In this work, we used the clinical data related to the DVPs to validate and score the proposed approaches. To visualize the clinical data related to the clustered DVPs, we employed the radar plot in Figure 7. These plots represent the average value of each clinical parameter for each cluster. From visual inspection it is clear that almost all the clustering approaches result in clusters which are associated with differences in clinical parameters. To quantify this, we first performed statistical tests to assess whether there were significant differences between the clinical data for each cluster. Significant differences were found between clusters obtained using all the approaches. To investigate which method is able to better differentiate the clinical data related to the clusters we implemented a modified version of the CSM approach (Boudaoud et al., 2010). For each clinical data contained in the dataset and for each method, we assessed the capability of the latter to cluster waves in a clinical relevant manner. Table 5 reports the obtained results. Depending on the considered data, the method that obtained the largest distance changes. When clustering with with the optimal approach, the distances are smaller. This finding is logic, since the starting support space is unaltered. The CNN and DDTW approaches (template and baseline) seem to always score large distances among all the clinical data. Age and transit time appear to have the largest difference, perhaps being the primary determinants for DVPs shape. Clusters appear to correspond to different clinical characteristics, and could provide insights into a subject’s vascular age as they are most strongly influenced by age and pulse transit time. This finding is in accordance with several studies (Alty et al., 2007; Brillante et al., 2008; Yousef et al., 2012). Physiologically, the aging process leads to increasing arterial stiffness (Bortolotto et al., 2000) which is reflected on the DVP shape as a less marked backward wave and dicrotic notch (Dawber et al., 1973). Arterial stiffness is directly correlated to the transit time. The more rigid the arteries, the smaller is the transit time due to a physiological loss of compliance of the arteries with the age (Nitzan et al., 2001). The obtained clusters seem to be able to differentiate among DVP related to subjects with different levels of arterial stiffening. The obtained clusters exhibit differences in dicrotic notch characteristics and also in the shape of the systolic portion of the DVP (see Figure 7). The systolic peak becomes wider with age and the diastolic wave disappears with age (Figure 6). However, further studies are needed to better understand the relation between the obtained clusters and their physiological meaning.

**TABLE 5:** PDF distances for each clinical data for each proposed method. The larger is the scored distance the better the method is able to cluster DVPs in a clinical relevant manner. The methods are scored, for each clinical data, from the best to the poorest.

Variable	Distance	Method	Variable	Distance	Method	Variable	Distance	Method
Age	0.19	CNN AE baseline	0.05	BMI	CNN AE template	0.05	BPM	CNN AE baseline
Age	0.18	DDTW baseline	0.05	BMI	CNN AE baseline	0.04	BPM	DDTW baseline
Age	0.17	CNN AE template	0.05	BMI	DDTW baseline	0.04	BPM	CNN AE template
Age	0.17	Handcrafted template	0.04	BMI	Handcrafted template	0.03	BPM	Handcrafted template
Age	0.17	Handcrafted baseline	0.04	BMI	Handcrafted baseline	0.03	BPM	Handcrafted baseline
Age	0.08	DDTW template	0.03	BMI	DDTW template	0.03	BPM	DDTW template
Age	0.07	Handcrafted optimal	0.02	BMI	Handcrafted optimal	0.02	BPM	Handcrafted optimal
Age	0.05	CNN AE optimal	0.01	BMI	CNN AE optimal	0.01	BPM	CNN AE optimal
Age	0.05	DDTW optimal	0.01	BMI	DDTW optimal	0.01	BPM	DDTW optimal
Height	0.06	Handcrafted baseline	0.08	PAD	CNN AE template	0.09	PAS	CNN AE baseline
Height	0.06	CNN AE baseline	0.08	PAD	CNN AE baseline	0.09	PAS	CNN AE template
Height	0.06	CNN AE template	0.07	PAD	Handcrafted template	0.08	PAS	DDTW baseline
Height	0.06	Handcrafted template	0.07	PAD	DDTW baseline	0.08	PAS	Handcrafted template
Height	0.05	DDTW baseline	0.06	PAD	Handcrafted baseline	0.07	PAS	Handcrafted baseline
Height	0.02	Handcrafted optimal	0.03	PAD	DDTW template	0.03	PAS	DDTW template
Height	0.02	DDTW template	0.03	PAD	Handcrafted optimal	0.03	PAS	Handcrafted optimal
Height	0.02	CNN AE optimal	0.02	PAD	CNN AE optimal	0.02	PAS	CNN AE optimal
Height	0.01	DDTW optimal	0.02	PAD	DDTW optimal	0.02	PAS	DDTW optimal
TT	0.17	DDTW baseline	0.09	PWV	DDTW baseline	0.05	Weight	DDTW baseline
TT	0.17	CNN AE baseline	0.09	PWV	CNN AE baseline	0.05	Weight	CNN AE template
TT	0.16	Handcrafted template	0.09	PWV	Handcrafted baseline	0.05	Weight	CNN AE baseline
TT	0.16	CNN AE template	0.09	PWV	CNN AE template	0.04	Weight	Handcrafted template
TT	0.15	Handcrafted baseline	0.08	PWV	Handcrafted template	0.03	Weight	DDTW template
TT	0.07	DDTW template	0.04	PWV	DDTW template	0.03	Weight	Handcrafted baseline
TT	0.06	Handcrafted optimal	0.03	PWV	Handcrafted optimal	0.02	Weight	Handcrafted optimal
TT	0.05	DDTW optimal	0.03	PWV	DDTW optimal	0.02	Weight	CNN AE optimal
TT	0.05	CNN AE optimal	0.03	PWV	CNN AE optimal	0.02	Weight	DDTW optimal

## 4 Conclusion

In this work we investigated several unsupervised approaches to cluster DVPs. We wanted to address whether or not a dataset composed of real DVPs can be described by 4 classes based on the dicrotic notch position, as previously reported by Dawber et al.

Our results indicate that DVP wave shapes do differ due to their dicrotic notch characteristics. However, there are additional differences such as width of the systolic peak and the strength of a secondary systolic wave. Investigating the optimal number of clusters with the help of methods such as inertia, silhouette score and prediction strength, we found 7 clusters of DVP wave shapes. Whilst these methods do require some subjective interpretation, using multiple methods allowed us to reduce the level of subjectivity.

The DDTW clustering approach performed best in this study, providing better separation between clusters than using either handcrafted features, or a CNN autoencoder approach. The DDTW approach takes into account the shape of the DVP wave by applying DTW to the first derivative of the DVP wave, and may therefore confer benefit over the previously proposed approach of applying DTW to the original DVP wave.

The different clusters of DVP waves correspond to different clinical characteristics. The clustering revealed that DVP wave shape was primarily associated with pulse transit time and age, which is in accordance with other studies Yousef et al. (2012); Alty et al. (2007); Brillante et al. (2008). Therefore, these clusters may provide insight into a subject’s vascular age. However, further studies are needed to better investigate the relationship between PWV and age and its effect on the DVP morphology. However, further studies are needed to better understand the relation between the obtained clusters and their physiological meaning.

Further improvements will focus on improving the measure of similarity *d* to assess differences in the probability density function by taking into consideration the distance between the averaged clinical values, the standard deviation and the variability among clusters. Other methods such as Gaussian and exponential modelling will be taken into consideration to extract relevant features from the DVP. In order to test the presented approach on a public dataset for comparison, it would be very helpful if public PPG datasets were created which contain PPG signals alongside reference cardiovascular measurements such as systolic blood pressure, pulse wave velocity and generic data such as age, weight and BMI.

## Data Availability

The datasets presented in this article are not readily available because they are private datasets. Requests to access the datasets should be directed to magid@axelife.com.
